# Cholesterol crystal formation is a unifying pathogenic mechanism in the development of diabetic retinopathy

**DOI:** 10.1007/s00125-023-05949-w

**Published:** 2023-06-14

**Authors:** Sandra S. Hammer, Tim F. Dorweiler, Delaney McFarland, Yvonne Adu-Agyeiwaah, Natalia Mast, Nicole El-Darzi, Seth D. Fortmann, Sunil Nooti, Devendra K. Agrawal, Irina A. Pikuleva, George S. Abela, Maria B. Grant, Julia V. Busik

**Affiliations:** 1 Department of Physiology, Michigan State University, East Lansing, MI USA; 2 Department of Ophthalmology and Visual Sciences, University of Alabama at Birmingham, Birmingham, AL USA; 3 Department of Ophthalmology and Visual Sciences, Case Western Reserve University, Cleveland, OH USA; 4 Department of Translational Research, Western University of Health Sciences, Pomona, CA USA; 5 Department of Medicine, Michigan State University, East Lansing, MI USA

**Keywords:** Blood retinal barrier, Cholesterol crystals, Complement activation, Cyclodextrin, Diabetic retinopathy, Endothelial cells, Fibrate, Hyper-reflective crystalline deposits, Inflammation, Statin

## Abstract

**Aims/hypothesis:**

Hyper-reflective crystalline deposits found in retinal lesions have been suggested to predict the progression of diabetic retinopathy, but the nature of these structures remains unknown.

**Methods:**

Scanning electron microscopy and immunohistochemistry were used to identify cholesterol crystals (CCs) in human donor, pig and mouse tissue. The effects of CCs were analysed in bovine retinal endothelial cells in vitro and in *db*/*db* mice in vivo using quantitative RT-PCR, bulk RNA sequencing, and cell death and permeability assays. Cholesterol homeostasis was determined using ^2^H_2_O and ^2^H_7_-cholesterol.

**Results:**

We identified hyper-reflective crystalline deposits in human diabetic retina as CCs. Similarly, CCs were found in the retina of a diabetic mouse model and a high-cholesterol diet-fed pig model. Cell culture studies demonstrated that treatment of retinal cells with CCs can recapitulate all major pathogenic mechanisms leading to diabetic retinopathy, including inflammation, cell death and breakdown of the blood–retinal barrier. Fibrates, statins and α-cyclodextrin effectively dissolved CCs present in in vitro models of diabetic retinopathy, and prevented CC-induced endothelial pathology. Treatment of a diabetic mouse model with α-cyclodextrin reduced cholesterol levels and CC formation in the retina, and prevented diabetic retinopathy.

**Conclusions/interpretation:**

We established that cholesterol accumulation and CC formation are a unifying pathogenic mechanism in the development of diabetic retinopathy.

**Graphical Abstract:**

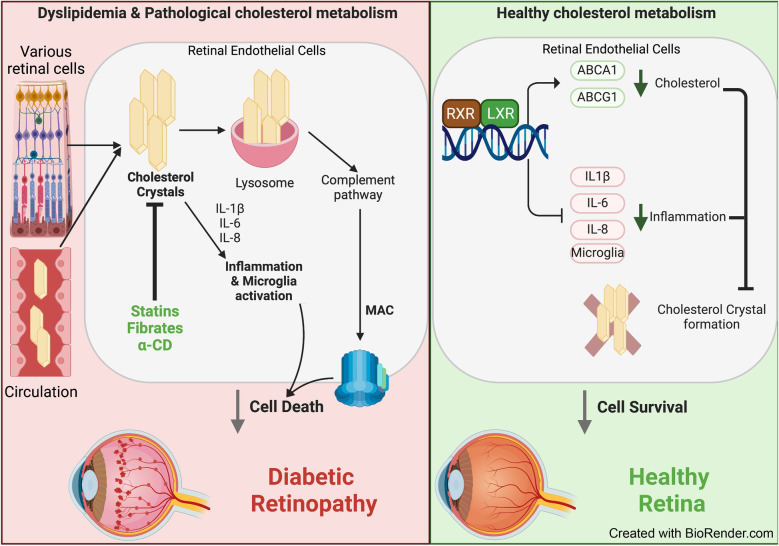

**Supplementary Information:**

The online version of this article (10.1007/s00125-023-05949-w) contains peer-reviewed but unedited supplementary material.


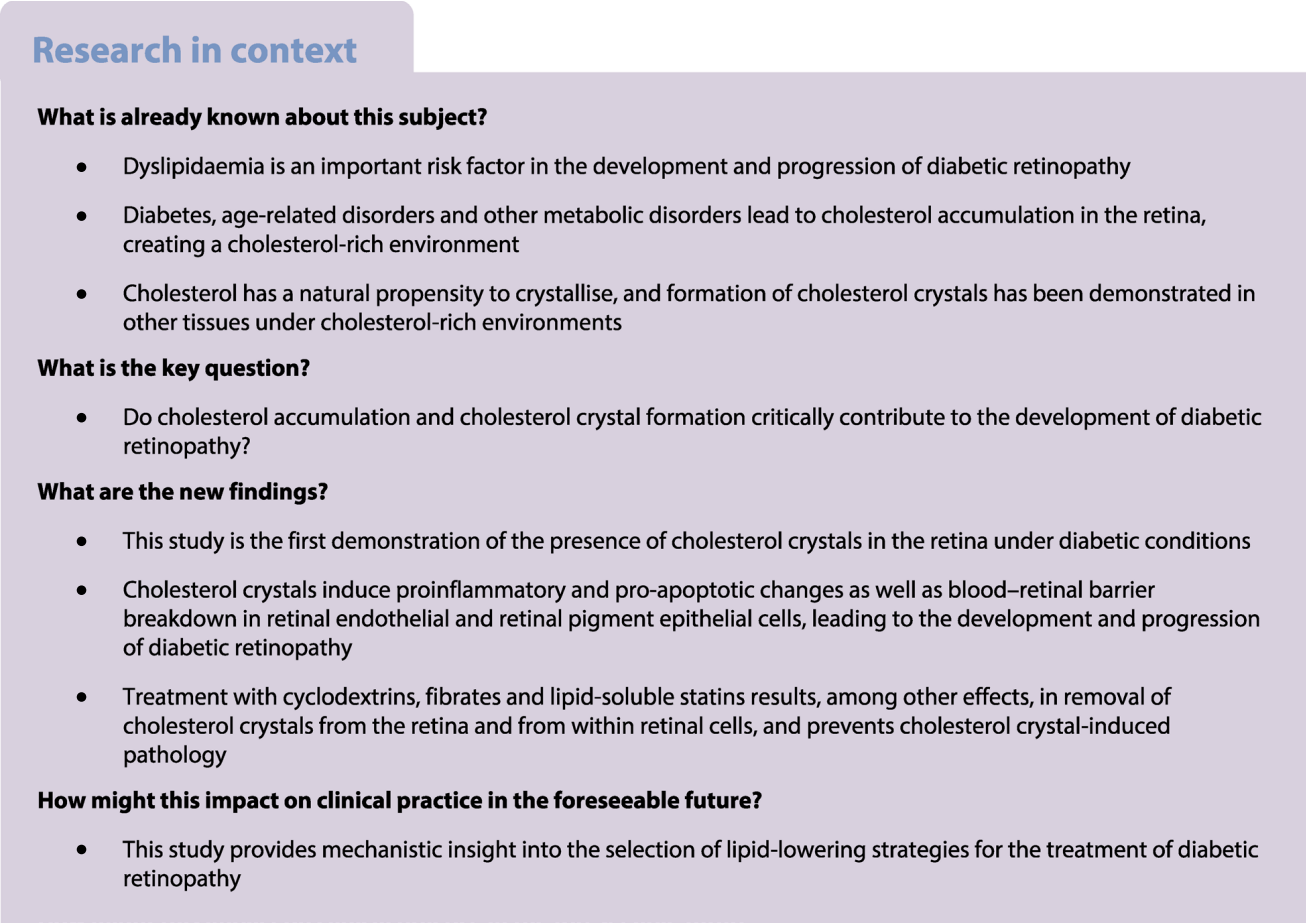


## Introduction

Cholesterol is a lipid that is indispensable for normal cellular, tissue and whole-organism function. Cholesterol production, transport and removal are tightly regulated. The retina is unique among peripheral organs due to the two barriers separating it from the systemic circulation [1]. The inner blood–retinal barrier (BRB) separates the retina from the intra-retinal vascular network. This barrier has tight junctions and is virtually impermeable to cholesterol when intact [2]. The outer BRB, which includes a layer of retinal pigment epithelial (RPE) cells and separates the retina from the choroidal circulation [3], is partially permeable to cholesterol [4]. Cholesterol input to the retina includes local biosynthesis and uptake of lipoprotein particles from the choroidal circulation at the basal membrane of RPE cells. Once in the RPE, cholesterol is effluxed both basolaterally and apically by ATP-binding cassette transporter 1 and ATP-binding cassette sub-family G member 1, and is complexed with apolipoproteins B and E, and other apolipoprotein B and E-containing particles, which deliver cholesterol either directly to the choroidal circulation by reverse cholesterol transport or to the neural retina [5]. In addition to reverse cholesterol transport, both RPE and neural retina metabolise cholesterol to more soluble oxysterols [6]. The oxysterols generated are the activating ligands for liver X receptors that control reverse cholesterol transport. Oxysterol production, activation of liver X receptor α and expression of ATP-binding cassette transporters are downregulated in the diabetic retina, leading to cholesterol accumulation and creating a cholesterol-rich environment similar to that found in atherosclerotic plaques [7]. The cholesterol-rich environment in atherosclerotic plaques has been previously shown to promote formation of cholesterol crystals (CCs) [8, 9]; however, CCs were suggested to be unique to the large blood vessels and a specific bifurcation point.

With the advancement of spectral-domain optical coherence tomography (OCT) imaging, several types of hyper-reflective deposits have been identified in retinal pathologies, including age-related macular degeneration (AMD) [10–14], Coats’ disease [15], atheroembolic disease [16], Bietti crystalline dystrophy and other crystalline retinopathies [17] and diabetic retinopathy [18, 19]. The origin and composition of hyper-reflective deposits varies depending on the size, shape, location within the retina and disease status. The hyper-reflective foci in AMD were identified as RPE cell remnants [14, 20, 21], and hyper-reflective foci at the vitreo-retinal interface in diabetic retinopathy were attributed to activated macrophages [19]. A particular type of hyper-reflective deposit, namely hyper-reflective crystalline deposits, have the appearance of crystals on OCT imaging [10–13, 15, 22–24]. There are several potential crystals that can form in biological tissue, mainly NaCl, calcium oxalate, calcium phosphate, monosodium urate and cholesterol. Based on the size and appearance of retinal hyper-reflective crystalline deposits in AMD, they were suggested to be composed of CCs [13]; however, direct composition analysis and assignment was not available. Our understanding of the composition and pathogenic nature of hyper-reflective crystalline deposits has been hampered by the lack of analytical tools to study CCs. While virtually insoluble in aqueous solutions, CCs are often overlooked in traditional scanning electron microscopy (SEM) and immunohistochemistry as they readily dissolve in the organic solvents used for tissue processing, thus masking potential involvement of CCs in pathogenic mechanisms [25]. CCs are recognised by the innate immune system as foreign bodies because of their shape, firmness and very low solubility in aqueous solutions [26], leading to activation of all three complement pathways (classical, lectin and alternative) in the extracellular space, formation of an intracellular complosome and accelerated cellular damage [27–29]. CCs can also induce inflammation via the NLR family pyrin domain-containing 3 (NLRP3) inflammasome that mediates formation of activated IL-1β, a key inflammatory mediator in diabetic retinopathy [27, 30].

The levels of HDL, the only known molecule in the body that can dissolve CCs, are often reduced in diabetes [31]. HDL also has poor access to deep tissue sites such as the retina. Thus, diabetic individuals are particularly at risk of developing CCs.

## Methods

### Animal experiments

All animal procedures were approved and monitored by the Institutional Animal Care and Use Committee at Michigan State University (IACUC # 201900370). Five-week-old male BKS.Cg-*Dock7*^*m*^ +/+ *Lepr*^*db*^/J were purchased from The Jackson Laboratory (https://www.jax.org/strain/000642). Mice heterozygous for *Lepr*^*db*/m^ (*db*/m) were used as controls, and mice homozygous for *Lepr*^*db/db*^ (*db*/*db*) were used as a model of diabetes. Upon arrival of the animals, blood glucose was tested daily and the mice were considered suitable as a model of diabetes if blood glucose levels were above 13.88 mmol/l at two consecutive measurements. After this initial confirmation of diabetes, blood glucose was tested monthly and HbA_1c_ was measured using a Mouse HbA_1c_ Assay Kit (80310; Crystal Chem, USA) at the completion of the study. After 6 months, a randomly assigned subgroup of diabetic animals was injected with 4 g/kg α-cyclodextrin (α-CD) (C4680; Sigma-Aldrich, USA) three times a week for 2 weeks. *db*/m, *db*/*db* and *db*/*db* + α-CD-treated animals were euthanised and retinal samples were collected. The samples were masked, and the experimenters were blind to group assignments and outcome assessment. The *db/db* animals that did not develop diabetes (blood glucose below 13.88 mmol/l) were excluded from the study. Retinal cholesterol input was measured in two separate experiments as described previously [32]: one used ^2^H_2_O and the other used ^2^H_7_-cholesterol (0.3% in peanut oil, 65 mg/ml) delivered by gavage. Calculations of retinal cholesterol biosynthesis and uptake rates are given in electronic supplementary material (ESM) Table 1.

A previously described Yucatan miniswine prediabetic high-cholesterol diet model [33] was used as detailed in the ESM Methods.

### Quantitative RT-PCR

RNA extraction and quantitative RT-PCR were performed as previously described [34]. The primer sequences are given in ESM Table 2.

### Cell culture and treatment

Human retinal endothelial cells (HREC) were isolated from tissue provided by the National Disease Research Interchange (USA) and EverSight Midwest Eye-Banks (USA) and cultured as previously described [7]. Detailed donor information has been provided previously [35] and a summary is provided in ESM Table 3. Passage 3–6 was used in this study. Bovine retinal endothelial cells (BREC) were isolated from bovine eyes and cultured as described previously [36]. Passages 4–8 were used in all studies. Pure CCs were made by dissolving 0.5 g cholesterol (C8667; Sigma-Aldrich, USA) in 100 ml methanol. Monohydrate CCs were made dissolving 0.5 g cholesterol (C8667; Sigma-Aldrich, USA) in 100 ml methanol and 5 ml water. Solutions were allowed to evaporate, and crystals were scraped and reconstituted in water at a concentration of 6 mg/ml. Crystal solutions were sonicated for 1 min at 35,000 Hz (Isonic, USA). A subset of cells was treated with 1 μmol/l rosuvastatin (SML1264; Sigma), 1 μmol/l atorvastatin (PHR1422; Sigma), 1 μmol/l fenofibrate (F6020; Sigma) or 10 mmol/l α-CD (C4680; Sigma) dissolved in culture medium.

### Cell death assays

Cell death was assayed using a trypan blue exclusion assay and Annexin V staining (ab14085; Abcam, UK). Apoptosis was thermally induced (50°C for 10 min) as a positive control.

### RNA sequencing

TRIzol reagent was added to cell lysate, and pure RNA was collected using the Zymogen Direct-zol RNA MiniPrep Kit (Zymo Research, USA). cDNA generation, library preparations and sequencing were performed by the University of Alabama Genomics Core Laboratory. Illumina paired-end 75 bp sequencing was used, with 25 million reads per sample. The programming language R [37] was used for analysing RNA-seq count data and ggplot2 [38] was used for visualisations. The raw sequencing data were aligned to the human reference genome (GRCh38) using the STAR method (Spliced Transcripts Alignment to a Reference) [39], and quant mode was used to generate raw transcript counts. DESeq2 [40] was used to identify differentially expressed genes (DEGs), which were defined as those with adjusted *p* values < 0.01. Complete lists of DEGs and pathways are supplied in ESM Tables 4 and 5.

### Scanning electron microscopy

Retinal tissue and Bruch’s membrane/choroid tissue (1×1 mm in size) from a non-diabetic donor (87-year-old white man), a diabetic donor without retinopathy (79-year-old white woman, duration of diabetes 10 years) and an insulin-dependent diabetic donor with proliferative diabetic retinopathy (PDR) (73-year-old white man, duration of diabetes not known) were isolated from the eyes, which had been fixed in 4% paraformaldehyde. The diabetic retinopathy status of each donor was ascertained based on agreement between medical records provided by the eye bank and postmortem fundus evaluation and OCT imaging of the donor eyes. The retinal/choroid tissue was from the periphery outside the macula.

SEM images were taken at the edge of individual pieces. Parts of the same tissue pieces were used for processed and unprocessed SEM samples. For processed samples, retinas were fixed in 1% osmium tetroxide in 0.1 mol/l sodium phosphate buffer for 1 h. Following a 30 min rinse in water, samples were dehydrated in an ethanol series (25%, 50%, 75%, 95%) for 15 min at each gradation, and with three 15 min changes in 100% ethanol at the end. Samples were critical point-dried in a model EM CPD300 critical point dryer (Leica Microsystems, Austria) using carbon dioxide as the transitional fluid. For unprocessed samples, retinas were subjected to vapour fixation with 2% osmium tetroxide for at least 48 h. Samples were mounted on aluminium stubs using high-vacuum carbon tabs (SPI Supplies, USA). Samples were coated with gold (approximately 30 nm thickness) in an Emscope sputter coater model SC500 sputter coater (Emzer, Spain) purged with argon gas. Samples were examined in a JEOL 6610LV scanning electron microscope with a tungsten hairpin emitter (JEOL, Japan).

### Immunocytochemistry

Tissue from the non-diabetic control donor and the PDR donor comprised Bruch’s membrane and choroid tissue, while that from the diabetic donor without diabetic retinopathy comprised the entire retina from the choroid to the photoreceptor layer. All donor tissues were obtained from the retinal periphery. Frozen sections (10 µm thickness) were stained using antibody against ionised calcium-binding adaptor molecule 1 (Iba1 ) (ab178846, Abcam) at a dilution of 1:250 overnight at 4°C, followed by chicken anti-rabbit Alexa Fluor 594 secondary antibody Bodipy FL C12 (C3927 MP, Sigma) at a dilution of 1:500 for 2 h at room temperature with nucleus counterstaining using DAPI (Sigma-Aldrich). For cell culture studies, HRECs were stained with antibody against membrane attack complex (MAC) C5b-9 (ab55811, Abcam) at a dilution of 1:100 in PBS with 1.5% BSA overnight at 4°C followed by incubation with chicken anti-rabbit Alexa Fluor 594 secondary antibody at a dilution of 1:500 for 2 h at room temperature and nucleus counterstaining using DAPI (Sigma-Aldrich). For tight junction visualisation, BRECs were stained with anti-ZO-1 antibody (ab59720, Abcam) at a dilution of 1:100, or anti-claudin-5 antibody (PA5-99415, Thermo Scientific) at a dilution of 1:100 in PBS with 1.5% BSA overnight at 4°C, followed by incubation with goat anti-rabbit Alexa Fluor 488 secondary antibody at a dilution of 1:500 in PBS with 1.5% BSA for 2 h at room temperature.

### In vitro permeability assay

BRECs were grown on 0.4 μm pore Transwell filters (Corning Costar, USA) for 24 h, treated with hydrocortisone for 36 h, and then with recombinant human vascular endothelial growth factor (VEGF)_165_ (50 ng/ml for 30 min), CCs (2 mg/ml for 24 h) and α-CD (1 μmol/l for 24 h). Paracellular permeability to 70 kDa RITC-dextran (Sigma-Aldrich) was measured after 24 h as previously described [41].

### Statistics

The number of animals to be included in each study was determined by a power calculation based on our previous results for the variables tested in the experiment. As we did not have prior data on CCs in the retina, the power calculation for CC counts was based on our data obtained in other organs [8]. Five animals per group were needed to detect 0.05 significance level with 0.92 power. Student’s paired *t* test was used to analyse data with two groups. In experiments with multiple group comparisons, one-way ANOVA with post hoc analysis using Tukey’s range test (GraphPad Prism 7, GraphPad Software, USA) was used. All values are expressed as means ± SD. *p* values < 0.05 were considered significant. DEGs in bulk RNA-sequencing data were identified using DESeq2 with default parameters. Adjusted *p* values were calculated using the false discovery rate method, with adjusted *p* values < 0.01 considered statistically significant.

## Results

**Fig. 1:**
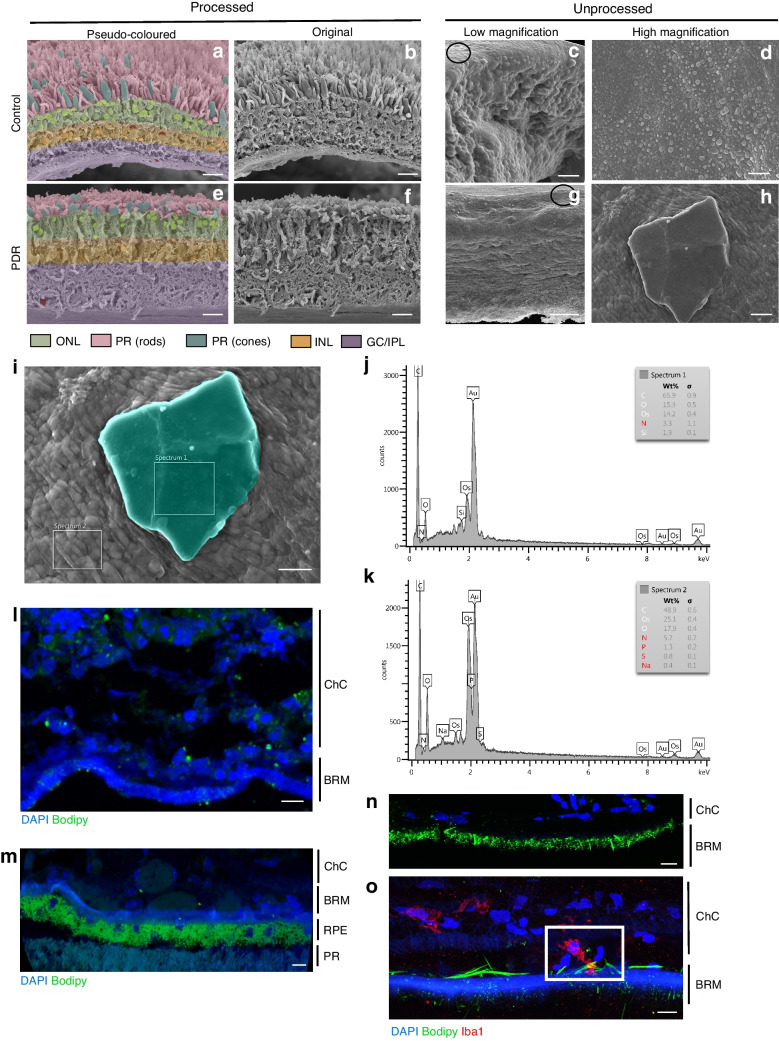
Increased retinal cholesterol levels and cholesterol crystallisation in vivo result in elevated inflammation, cell death and vascular permeability*.* (**a**–**h**) Pseudo-coloured and non-coloured processed and unprocessed tissue samples from a non-diabetic control donor and a diabetic donor with PDR. A representative CC is shown in (**h**) for the diabetic donor with PDR. Black circles in (**c**) and (**g**) indicate the general vicinity of the areas where the high-magnification images were taken. (**i**–**k**) EDS composition analysis of a representative crystal (pseudo-coloured in teal) and surrounding tissue from the PDR retinal section. (**l**, **m**) Tissue samples from a non-diabetic control donor (**l**) and a diabetic donor without diabetic retinopathy (**m**) stained with Bodipy (green) and DAPI (blue). (**n**, **o**) Proliferative diabetic choroid from a diabetic donor with PDR stained with Bodipy (green), DAPI (blue) and Iba1 (red). Scale bars: 50 µm (**a**, **b**, **e**, **f**), 20 µm (**c**, **g**), 10 µm (**d**, **h**, **i**), 50 µm (**l**, **m**, **n**), 15 µm (**o**). BRM, Burch’s membrane; ChC, choroidal capillaries; GC/IPL, ganglion cell inner plexiform layer; INL, inner nuclear layer; ONL, outer nuclear layer; PR, photoreceptors; RPE, retinal pigment epithelium

The ultrastructure of human retinas from a donor without diabetes (Fig. 1a–d) and a donor with PDR (Fig. 1e–h) was first determined using traditional SEM analysis. Original images are shown in Fig. 1b and f; pseudo-coloured images of the same sections are shown in Fig. 1a and e. Using fully processed traditional SEM images as a guide, SEMs of the neighbouring tissues from control and PDR retinas were prepared without organic solvents (or unprocessed) to determine the presence and location of CCs. Using preparation without organic solvents, which preserves lipids at the expense of structural details, we observed the presence of high-lipid-environment ‘lipid pools’ (opaque white specks at the top of Fig. 1g) and formation of CCs in the neural retina of the human donor with PDR (Fig. 1h), but not in the retina of the control donor (Fig. 1d). Using energy-dispersive X-ray spectroscopy (EDS), we performed elemental analysis of the crystals and neighbouring tissues (Fig. 1j,k). The analysis showed high carbon and oxygen content and a lack of other elements, consistent with the crystal being a CC, rather than NaCl, calcium oxalate, calcium phosphate or monosodium urate. The results obtained in human PDR donor tissue were further strengthened by study of a diabetic mouse model and prediabetic pig model (ESM Methods and ESM Fig. 1). Moreover, C12 Bodipy staining revealed the presence of CCs in the Bruch's membrane of human PDR choroidal sections (Fig. 1n), but not in the Bruch's membrane of the control donor or the diabetic donor without retinopathy (Fig. 1l,m). Both needle-like pure CCs and blade-like monohydrate CCs were observed. Co-staining with macrophage/microglia marker Iba1 revealed activated microglia engulfing CC (Fig. 1o).

**Fig. 2:**
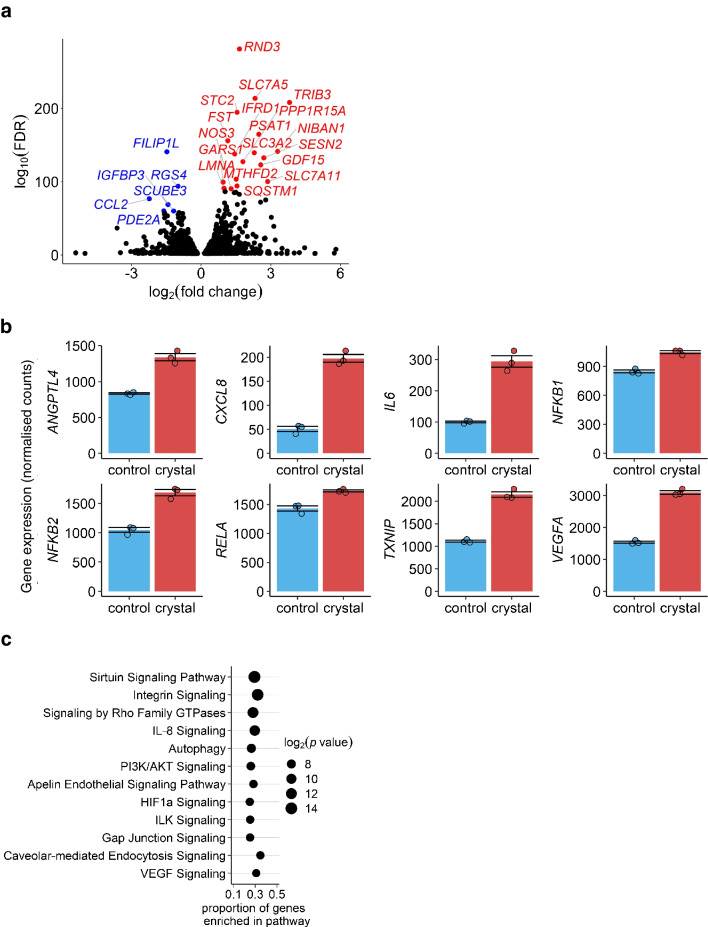
Bulk RNA sequencing of HREC treated with CC in vitro. HREC were treated with pure CCs (2 mg/ml) for 24 h and analysed by RNAseq. (**a**) Volcano plot showing statistically significant (adjusted *p* value <0.01) DEGs in HREC treated with CCs (*n*=3) vs vehicle control (*n*=3). The labelled genes in the volcano plot are DEGs with the lowest adjusted *p* values. For upregulated DEGs (labelled in red), an adjusted *p* value threshold of <1×10^–90^ was used; for downregulated DEGs (labelled in blue), an adjusted *p* value threshold of <1×10^–60^ was used. (**b**) Mean normalised expression levels of selected DEGs implicated in diabetic retinopathy (*n*=3 biological replicates per group). (**c**) A total of 2573 genes identified as DEGs (1285 upregulated and 1288 downregulated) were used in pathway analysis. The pathways shown were selected from the top 45 most significant pathways. FDR, false discovery rate; HIF1a, hypoxia inducible factor 1 subunit alpha; ILK, integrin-linked kinase; PI3K, phosphatidylinositol 3-kinase; VEGF, vascular endothelial growth factor

In addition to the outer retina and Bruch’s membrane as described above, CCs present in the circulation are likely to affect the inner BRB through CC embolism. We next determined the effect of CCs on HREC. Cells were treated with 2 mg/ml pure CC for 24 h for subsequent RNAseq analysis to determine global CC-induced changes in HREC. A Volcano plot for DEGs is shown in Fig. 2a. Selected genes with the lowest adjusted *p* values are labelled. Pathway analysis using all DEGs is shown in Fig. 2c; mean normalised expression levels of selected DEGs implicated in diabetic retinopathy are shown in Fig. 2b.

**Fig. 3:**
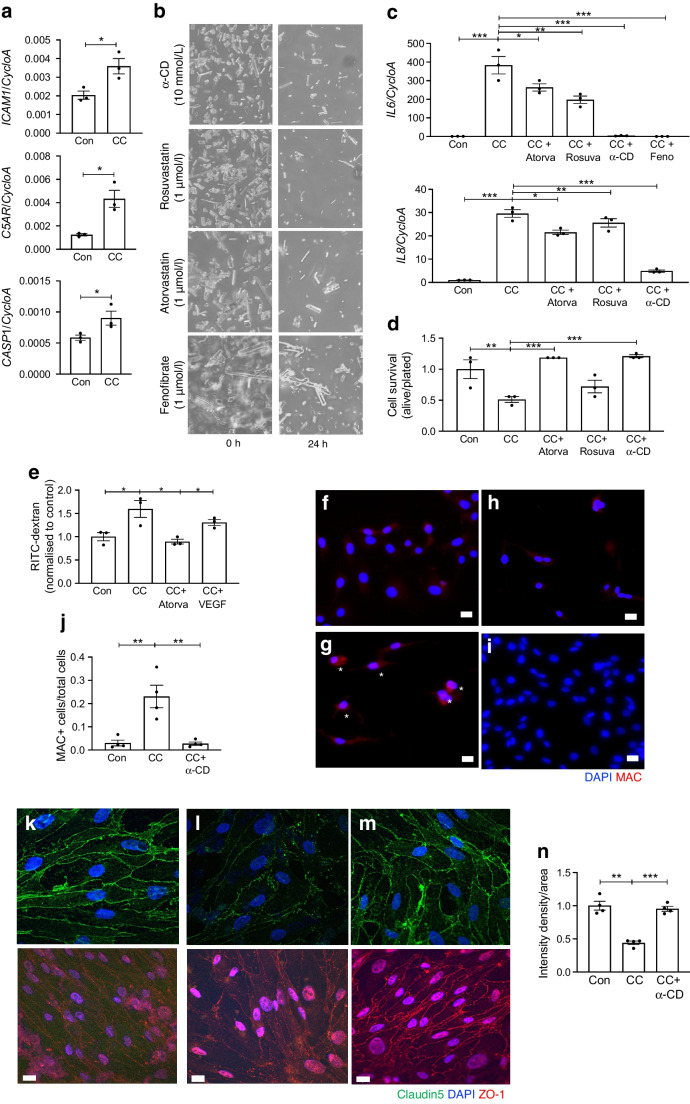
CC-induced inflammation, cell death and breakdown of the BRB. HREC (**a–j**) or BREC (**k–n**) were treated with CC (2 mg/ml) or crystals pre-treated for 1 h with atorvastatin (1 μmol/l), rosuvastatin (1 μmol/l), fenofibrate (1 μmol/l) or α-CD (10 mmol/l). (**a**) As determined by quantitative RT-PCR, CC treatment results in elevated *ICAM1*, *C5AR1* and *CASP1* mRNA expression. Cyclophilin A (*CycloA*; also known as *PPIA*) was used as a housekeeper. (**b**) Ex vivo images of CC before (0 h) and after (24 h) administration of the cholesterol-dissolving drugs. (**c**) After 24 h, *IL6* and *IL8* mRNA expression was measured. (**d**) Effect of atorvastatin and VEGF on CC-induced increase in permeability to RITC-dextran. (**e**) Cell death as measured by the trypan blue exclusion assay. (**f**, **g**) The MAC (red) is activated in CC-treated HREC cells (**g**) when compared with control cells (**f**). MAC-positive cells are indicated by asterisks. (**h**) Treatment with α-CD (10 μmol/l) prevents MAC formation in HREC. (**i**) Control cells treated with α-CD. (**j**) Number of MAC-positive cells under the various treatments. (**k**–**m**) Treatment with α-CD (10 mmol/l) prevented CC-induced border disruption in BREC: claudin-5 (green) and ZO-1 (red) staining in control cells (**k**), cells treated with CC (**l**) and cells treated with α-CD plus CC. (**n**) Quantification of staining intensity. *n*=3–4 biological replicates per group. Data were analysed by one-way ANOVA followed by Dunnett’s multiple comparison test (**c**, **d**, **e**, **j**, **n**) or two-tailed, unpaired Student's *t* test (**a**), *p<0.05, **p<0.01, ***p<0.001. Scale bars, 10 µm. Atorva, atorvastatin; Con, control; Feno, fenofibrate; Rosuva, rosuvastatin

Selected DEGs were further analysed by quantitative RT-PCR. A significant increase in expression of *ICAM1* (Fig. 3a), *IL6* and *IL8* (Fig. 3c), proinflammatory markers whose levels are known to be increased in the diabetic retina, was observed. *CASP1* (encoding caspase-1, an inflammasome activator), was also significantly elevated as a result of CC administration (Fig. 3a). Due to the established role of CCs in complement activation, and the emerging evidence linking the complement system to progression of diabetic retinopathy, expression of the gene encoding complement receptor, *C5AR1*, and formation of MACs were analysed in CC-treated HREC. CC administration led to increased *C5AR1* mRNA expression (Fig. 3a) and elevated MAC immunocytochemical staining (Fig. 3g). Moreover, CC induced endothelial cell death (Fig. 3e).

As BRB breakdown is one of the first pathogenic changes in the diabetic retina, we next determined the effect of CCs on endothelial cell permeability and tight junction integrity. The tight junctions and vascular permeability experiments were performed using BREC. BREC grown to form a confluent monolayer were treated with CCs or vehicle for 24–48 h. CC treatment induced an increase in permeability that was significantly higher than that in control cells and cells treated with VEGF (50 ng/ml) (Fig. 3d). To gain mechanistic insight, the effect of CCs on tight junction integrity was determined by immunostaining for the tight junction proteins claudin-5 and ZO-1. Immunostaining revealed that CCs disrupted continuous immunostaining of claudin-5 and ZO-1 at the cell border (Fig. 3k,l).

Next, treatments to counteract the detrimental effects of diabetes were investigated. As fenofibrate was effective as a retinopathy treatment in clinical trials [42, 43], the effect of fenofibrate on CC-induced cell damage was investigated. Administration of fenofibrate significantly prevented CC-induced upregulation of inflammation (Fig. 3c). Remarkably, fenofibrate was more effective at dissolving CCs and preventing CC-induced damage than the lipid-sequestering agent α-CD (Fig. 3b,c). Treatment with cholesterol-lowering statins was also effective but less so than treatment with fenofibrate and α-CD (Fig. 3b–e). In addition to the effects on inflammation (Fig. 3c), cell death (Fig. 3e) and MAC activation (Fig. 3f–h), α-CD also prevented breakdown of retinal cell membrane integrity and restored vascular barrier function (as shown in Fig. 3d,k–n).

**Fig. 4:**
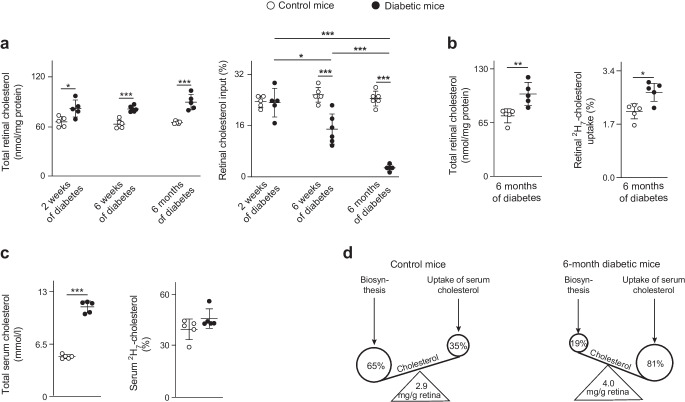
Diabetes disturbs retinal cholesterol homeostasis and affects retinal cholesterol input in a mouse model of type 2 diabetes. (**a**) Retinal cholesterol content and input in *db*/*db* mice vs non-diabetic mice after 2 weeks, 6 weeks and 6 months of type 2 diabetes. Input was assessed within 14 days by ^2^H incorporation into retinal cholesterol through drinking ^2^H_2_O. (**b**) Retinal cholesterol content and uptake in *db*/*db* mice vs non-diabetic mice after 6 months of type 2 diabetes. The uptake was assessed within 13 days by the incorporation of dietary ^2^H_7_-cholesterol into retinal cholesterol. (**c**) Total and ^2^H_7_-cholesterol in the serum of the diabetic mice. (**d**) Schematic summary of the relative contributions of retinal cholesterol biosynthesis and retinal cholesterol uptake from the systemic circulation to the total retinal cholesterol input in control mice and *db*/*db* mice after 6 months of diabetes. Data were analysed by one-way ANOVA with Tukey’s multiple comparison test (**a**) or by two-tailed, unpaired Student's *t* test (**b**, **c**). *n*=5 animals per group. *p<0.05, **p<0.01, ***p<0.001

BRB breakdown enables more cholesterol-containing apolipoprotein particles from the systemic circulation to enter the retina and potentially disturb retinal cholesterol homeostasis. In the next part of the study, we provided *db*/*db* mice with ^2^H-labelled water (^2^H_2_O) for 2 weeks before killing, and quantified the retinal cholesterol content after 2 weeks, 6 weeks and 6 months. We also measured total retinal cholesterol input (the sum of local retinal cholesterol biosynthesis and uptake from circulation) (Fig. 4a and ESM Table 1). The total retinal cholesterol levels were increased in all diabetic mice compared with controls, but these levels did not increase with diabetes progression. However, diabetes duration did have a significant effect on total retinal cholesterol input, which was reduced 1.7- and 9-fold after 6 weeks and 6 months of diabetes, respectively. This finding prompted us to perform a study on mice that had diabetes for 6 months, in which unlabelled 0.3% dietary cholesterol was replaced with ^2^H-labelled (^2^H_7_) cholesterol 13 days before the animals were killed. We measured total and retinal ^2^H_7_-cholesterol as well as total and serum ^2^H_7_-cholesterol (Fig. 4b,c). Using the data from the ^2^H_2_O experiment, we then calculated the relative contributions of retinal cholesterol biosynthesis and retinal uptake of systemic cholesterol to total retinal cholesterol input (ESM Table 1). The results suggested that 6 months of diabetes increased the retinal uptake of systemic cholesterol 2.3-fold (from 35% to 81%) and reduced retinal cholesterol biosynthesis 3.4-fold (from 65% to 19%) (Fig. 4d), probably as a compensatory response. Nevertheless, the total retinal cholesterol content remained increased 1.4-fold (4 vs 2.9 mg/g retina) after 6 months of diabetes. Thus, increased endothelial cell permeability and BRB breakdown in diabetes probably contribute to disturbance of cholesterol homeostasis in the retina.

**Fig. 5:**
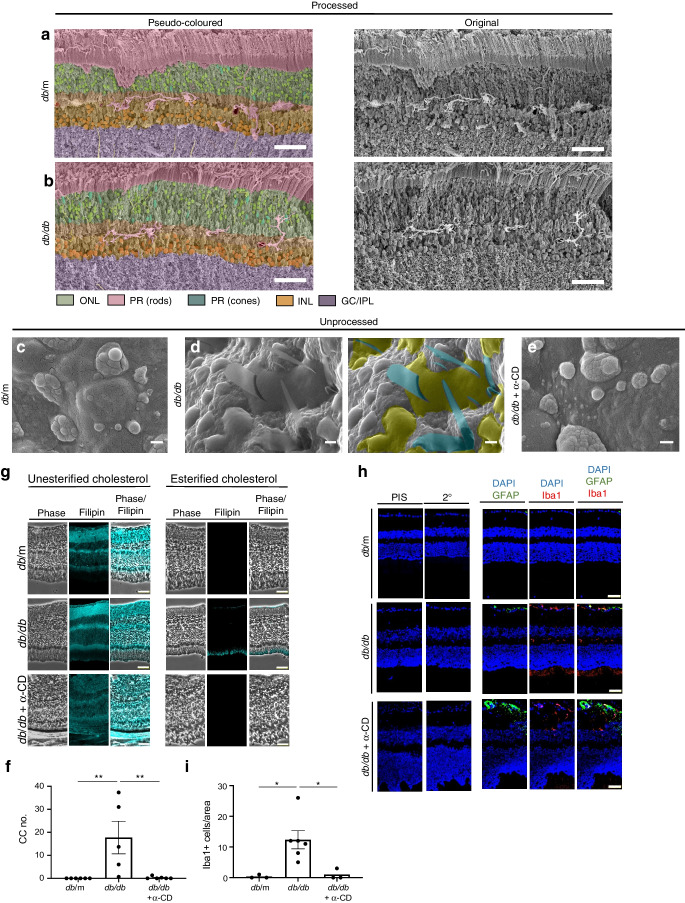
α-CD dissolves CCs and reduces Iba1 content in vivo*.* Representative pseudo-coloured and non-coloured processed SEM images for control mice (**a**) and 6-month-old diabetic (*db*/*db*) mice (**b**). Unprocessed SEM images for *db*/m mice (**c**), *db*/*db* mice after 6 months of diabetes (**d**) and *db*/*db* after 6 months of diabetes treated with α-CD (0.4 kg/ml) (**e**). α-CD was administered orally within the feeding water after 4 months of diabetes for two months. The right-hand image in (**d**) is a pseudo-coloured image highlighting CCs (teal) and lipid pools (olive) in unprocessed SEM of *db*/*db* retinas. (**f**) Quantification of the number of CCs; *n*=5–6 animals per group. (**g**) Phase and filipin staining of *db*/m, *db*/*db* and *db*/*db* + α-CD retinas. (**h**) Pre-immune serum (PIS), secondary antibody only control (2°), glial fibrillary acidic protein (GFAP) (green), Iba1 (red) and DAPI (blue) staining in *db*/m, *db*/*db* and *db*/*db* α-CD retinas. (**i**) Quantification of Iba1-positive cells; *n*=3–5 animals per group. Data were analysed by One-Way ANOVA followed by Dunnett’s multiple comparison test, *p<0.05, **p<0.01. Scale bars, 50 µm (**a**, **b**), 5 µm (**c**–**e**) or 50 µm (**g**, **h**). GC/IPL, ganglion cell inner plexiform layer; INL, inner nuclear layer; ONL, outer nuclear layer; PR, photoreceptors

As the lipid-sequestering agent α-CD was effective at CC removal in situ and in vitro, we next determined the effect of α-CD in vivo on CC formation in the retina of *db*/*db* mice. A significant increase in the amount of CCs was found in retinas of mice with diabetes of 6 months duration (Fig. 5c–e). As with human tissue (Fig. 1), CCs were detected in or around areas of lipid accumulation between the RPE and photoreceptor layers (Fig. 5d). No crystals were detected in similar areas of control retinas (Fig. 5c). These crystal structures were also identified in retinas of pigs fed a high-cholesterol diet (ESM Fig. 1). Strikingly, administration of α-CD for 2 months successfully reduced both unesterified and esterified cholesterol levels (Fig. 5g), removed CCs from diabetic mouse retinas (Fig. 5e,f), and reduced the inflammatory phenotype as indicated by microglia activation (Fig. 5h,i).

## Discussion

The presence of CCs is considered a hallmark trait of atherosclerotic plaques [44], but there is very limited information on CC formation in other tissues. Multiple types of hyper-reflective structures were recently identified by spectral-domain OCT imaging in the retina and choroid in retinal pathologies, including AMD [10–14], Coats’ disease [15], atheroembolic disease [16], Bietti crystalline dystrophy and other crystalline retinopathies [17] and diabetic retinopathy [18, 19]. The presence of hyper-reflective structures has also been proposed to be a novel prognostic biomarker in diabetic macular oedema [10–12, 22]. The origin and composition of hyper-reflective deposits varied depending on the size, shape, location within the retina and disease status. The hyper-reflective foci in AMD were identified as RPE cell remnants [14, 20, 21], while the hyper-reflective foci at the vitreo-retinal interface in diabetic retinopathy were attributed to activated macrophages [19]. A particular hyper-reflective structure, hyper-reflective crystalline deposits, are suggested to be comprised of CCs; however, these structures cannot be positively identified as CCs solely by spectral-domain OCT imaging, thus the identity of these crystals remains unknown [10–13, 15, 22–24]. Moreover, CCs are dissolved by the organic solvents that are traditionally used in tissue preparation for light and electron microscopy. Due to the ability of ethanol to dissolve lipid-based crystals, retinas were left unprocessed for CC identification in this study [25]. Crystalline structures that can form in biological tissues mainly include NaCl, calcium oxalate, calcium phosphate, monosodium urate and cholesterol. To determine the identity of crystalline structures observed in diabetic retinas, we performed elemental analysis of surfaces in SEMs using EDS. The electron beam directed at the SEM specimen can only penetrate to a depth of 0.02–1.0 μm, thus the tissue underneath the crystal does not affect the signal. Moreover, we have previously demonstrated [45] that EDS and Fourier transform infrared absorption spectra provided identical composition analysis for CCs found in human tissues and synthetic CCs, further confirming that EDS can be used for CC composition analysis. In this study, EDS analysis revealed compounds containing C and O in the crystals identified, providing strong support that the crystals are comprised of cholesterol rather than other potential naturally occurring crystalline structures. Combining modified SEM and confocal microscopy, we were able to demonstrate the presence of CCs in the retina of a diabetic donor with PDR. Although detection of CCs in the retina of a diabetic donor with PDR was important for this proof-of-concept study, future well-powered clinical trials are required to fully appreciate the role of CCs in progression of diabetic retinopathy and the therapeutic potential of targeting CCs in patients with diabetic retinopathy. We detected CCs in both the outer retina and Bruch’s membrane. CCs are probably present in other locations in the retina that are not accessible for SEM detection. Moreover, the retina is a cholesterol-rich tissue, and cholesterol staining in most layers would mask CCs even if they were present in the tissue. Indeed, CCs were previously detected by dissolving lipids and looking at the clefts left by crystals in the tissues [13]. However, this technique can only be used for large crystals. We were able to detect CCs in the Bruch’s membrane using Bodipy because there is very little cholesterol present in Bruch’s membrane, making detection of crystals possible. Although not detectable by the methods used in this study, CCs showered from atherosclerotic plaques into the circulation can cause retinal CC embolism, or atheroembolic disease [16], affecting inner retinal capillaries. To investigate the role of CCs in endothelial cell pathology in diabetic retinopathy, we performed mechanistic cell culture studies using HREC and BREC. Treatment of endothelial cells with CCs recapitulated key pathogenic events associated with the development of diabetic retinopathy. These include disruption of tight junctions, increased retinal endothelial cell permeability, BRB breakdown, proinflammatory changes, complement activation and endothelial cell death [46]. Interestingly, fenofibrate, which has shown promise in clinical trials for treatment of diabetic retinopathy independent of its effects on circulating lipids, was able to dissolve CC in vitro and prevent CC-induced pathology, similarly to the lipid-sequestering agent α-CD [47]. Fenofibrate was shown to prevent inflammatory changes, effects on retinal vascular permeability, and formation of acellular capillaries in diabetic retinopathy animal models [48, 49]. As the mechanism of fenofibrate therapeutic action in diabetic retinopathy is not known, the effect of fenofibrate on CCs provides clinically significant insight. Although less effective than fenofibrate and α-CD, several statins also dissolved CCs and reduced CC-induced pathology in endothelial cells.

Little is known about the conditions necessary for cholesterol crystallisation. Using ^2^H_2_O and ^2^H_7_-cholesterol, we showed that retinal uptake of systemic cholesterol was significantly higher in diabetic mice vs non-diabetic animals, and the former also showed an increase in serum cholesterol (Fig. 4c). Previously, we showed that cholesterol export out of the retina is impaired due to downregulation of reverse cholesterol export pathways such as ATP-binding cassette transporter 1/ATP-binding cassette sub-family G member 1 [34]. Thus, increased retinal cholesterol uptake and decreased cholesterol output lead to the elevated cholesterol levels in the retina, and create an ideal environment for crystal formation. In addition to the human diabetic retina and a type 2 diabetes mouse model, we identified CCs in a prediabetic pig model (pigs fed a high-cholesterol diet), demonstrating that CC accumulation is ubiquitous in several models with dysregulated cholesterol metabolism and high levels of cholesterol accumulation in the retina.

In the retina, CCs were abundant in high-cholesterol-containing areas at the RPE and photoreceptor interface. Due to the rapid turnover of photoreceptor outer segments and the phagocytotic nature of the RPE, this area of the retina is subject to highly dynamic lipid flow and recirculation. This highly turbulent area provides an ideal environment for excess cholesterol to accumulate and crystallise, similar to the tumultuous milieu in an atherosclerotic plaque, a common site of CC formation [8, 26]. Whether CCs originate in the retina in highly lipid dense areas or are mainly transported to the retina via the blood remains an active area of investigation. CCs may be released into the blood stream upon rupture of atherosclerotic plaques, potentially causing damage to distant sites such as the retina [8]. Confocal images of diabetic human choroidal sections reveal the presence of CC-like structures in Bruch’s membrane (Fig. 1o,p). This membrane is the innermost layer of the choroid, a vascular layer of the eye that provides nutrients and oxygen to the outer layers of the retina [50, 51]. Identifying CCs in this membrane suggests potential transport of crystals between the choroidal circulation and the RPE/photoreceptor layers.

To determine whether CC removal could have therapeutic value, we treated diabetic mice with the lipid-sequestering agent α-CD. Treatment with α-CD for 2 weeks led to CC removal and reduction of inflammatory markers, suggesting that not only prevention strategies but also intervention treatment strategies to remove CCs may be used for diabetic retinopathy.

This study demonstrated the presence and pathogenic nature of CCs in diabetic retinas, showing that CCs can activate all key pathogenic events in the diabetic retina. Strategies for correcting retinal cholesterol imbalances and/or removal of CCs may have therapeutic value in treatment of diabetic retinopathy.

## Supplementary Information

Below is the link to the electronic supplementary material.

Supplementary file1 (PDF 413 KB)

Supplementary file2 (XLSX 177 KB)

## Data Availability

The authors confirm that the data supporting the findings of this study are available within the article and its supplementary materials. RNAseq data are available in ESM Tables 4 and 5.

## Abbreviations

AMD: Age-related macular degeneration
BRB: Blood–retinal barrier
BREC: Bovine retinal endothelial cells
C5aR: Component 5a receptor 1
α-CD: α-Cyclodextrin
CC: Cholesterol crystal
DEG: Differentially expressed gene
EDS: Energy-dispersive X-ray spectroscopy
HREC: Human retinal endothelial cells
Iba1: Ionised calcium-binding adaptor molecule 1
MAC: Membrane attack complex
OCT: Optical coherence tomography
PDR: Proliferative diabetic retinopathy
RPE: Retinal pigment epithelium
SEM: Scanning electron microscopy
VEGF: Vascular endothelial growth factor
