# Supplementary material for: Cholesterol crystal formation is a unifying pathogenic mechanism in the development of diabetic retinopathy
Source: Diabetologia. 2023 Jun 14;66(9):1705–18. doi: 10.1007/s00125-023-05949-w (PMC10390399; doi:10.1007/s00125-023-05949-w)
Supplement: Supplementary file 1 — Supplementary file1 (PDF 413 KB) [file 125_2023_5949_MOESM1_ESM.pdf]

**ESM Methods**

Two to three months old female Yucatan miniswine were kept at the animal facility at Western University of Health Sciences, Pomona, CA with 12 hours light and dark cycle at a temperature range of 72°-74° F. The miniswine with similar body weights were divided randomly into two groups: control and high cholesterol diet right after weaning. The control swine were fed with the Mini-Pig Grower Diet (Test Diet # 5801) high cholesterol group were fed 1–1.5 lb/ animal/day special high cholesterol diet (Harlan Laboratories, Denver, CO). The major ingredients in the diet include: Corn 37.2.% corn (8.5% protein), 23.5% soybean meal (44% protein), 20% chocolate mix, 5% alfalfa, 4% cholesterol, 4% peanut oil, 1.5% sodium cholate, and 1% lard[1]. Both groups were allowed to drink water *ad libitum*. A total of 8 animals, 4 control and 4 animals on high cholesterol diet for 12-14 months were used in the study. Animal work was performed per the guidelines of the National Institutes of Health and USDA for the care and use of experimental animals. The animal research protocol No. R20IACUC038 of this study was approved by the Institutional Animal Care and Use Committee of the Western University of Health Sciences, Pomona, California. After the animals were euthanized, the eyes were enucleated and fixed in 4% paraformaldehyde before processing for SEM analysis. Cone-rich area centralis was used in the study.

**ESM Table 1.** Calculations of retinal cholesterol biosynthesis and uptake rates in non-diabetic (Con) and diabetic (Db) mice after 6 months of diabetes.

| Parameter | Serum | | Retina | |
|---|---|---|---|---|
| | Con | Db | Con | Db |
| Cholesterol content (mg/dL, serum; nmol/mg protein, retina) | 190 | 426 | 73 | 99 |
| Cholesterol content (mg/g wet tissue) | | | 2.9 | 4.0 |
| **Treatment with dietary $D_7$-cholesterol** | | | | |
| $D_7$-cholesterol fraction, 13 days (%) | 39.4 | 45.6 | 2.1 | 2.7 |
| Retinal cholesterol uptake, 13 days (%) | | | 5.3 | 5.9 |
| **Treatment with $^2$HO** | | | | |
| Total cholesterol input, 14 days (%) | | | 24.8 | 2.9 |
| Deuterium cholesterol enrichment from serum uptake, 14 days (%) | | | 14.1 | 1.4 |
| Tissue cholesterol biosynthesis, 14 days (%) | | | 10.7 | 1.5 |
| **Summary data** | | | | |
| Absolute rate of cholesterol input (μg/day/g wet tissue (%)) | | | 34.0 (100%) | 22.6 (100%) |
| Local biosynthesis (μg/day/g wet tissue (%)) | | | 22.1(65%) | 4.4 (19%) |
| Uptake from blood (μg/day/g wet tissue (%)) | | | 11.9 (35%) | 18.2(81%) |

**ESM Table 2**. Primer sequences for real-time PCR analysis.

| Species | Primer name | Forward (sense) | Reverse (anti sense) | NCBI ID# |
|---|---|---|---|---|
| **Human** | *ABCA1* | GGTGGTGTTCTT CCTCATTACT | CCGCCTCACATCTTC ATCTT | NM_005502.4 |
| | *ABCG1* | CTCCGCCTCATT GCCTATTT | GAAGATTAGACACTG TGGCCG | NM_016818.3 |

| | | | | |
|---|---|---|---|---|
| | *IL-6* | GGAGACTTGCCT GGTGAAA | GAGTAGTGAGGAAC AAGCCAG | NM_000600.5 |
| | *IL-8* | CTTGGCAGCCTT CCTGATTT | GGGTGGAAAGGTTT GGAGTATG | NM_000584.4 |
| | *C5aR* | TCTACTCACGCT CACGATTTG | GGTAGGGCAACCAG AAGATAAA | NM_001736.4 |

**ESM Table 3**. Characteristics of Donor

| | |
|---|---|
| Number of donors | 1 |
| Gender (M/F) | M |
| Age (years) | 44 |
| Ethnicity | Hispanic |
| Diabetes duration | No diabetes |
| Drug use | No |
| Alcohol use | No |
| Tobacco use | No |

**ESM Fig. 1**. Unprocessed SEM images of control (left) and high fat fed (middle) pig retinas after 12-14 months of High Fast Diet (HFD). Quantification shown on the right. N=4 per group. Two tailed Students T-Test, * p<0.05.

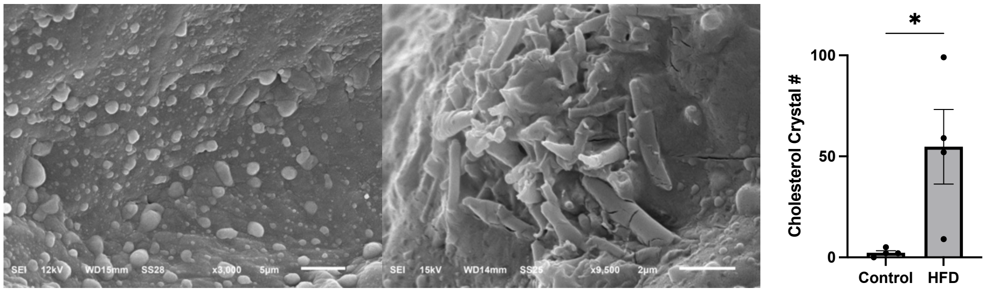



1 Gupta, G. K. *et al.* Suppressor of cytokine signaling-3 and intimal hyperplasia in porcine coronary arteries following coronary intervention. *Exp Mol Pathol* **91**, 346-352 (2011). https://doi.org:10.1016/j.yexmp.2011.04.004
